# Supplementary figures and images for: Bacterial killing by complement requires direct anchoring of membrane attack complex precursor C5b-7
Source: PLoS Pathog. 2020 Jun 22;16(6):e1008606. doi: 10.1371/journal.ppat.1008606 (PMC7351214; doi:10.1371/journal.ppat.1008606)

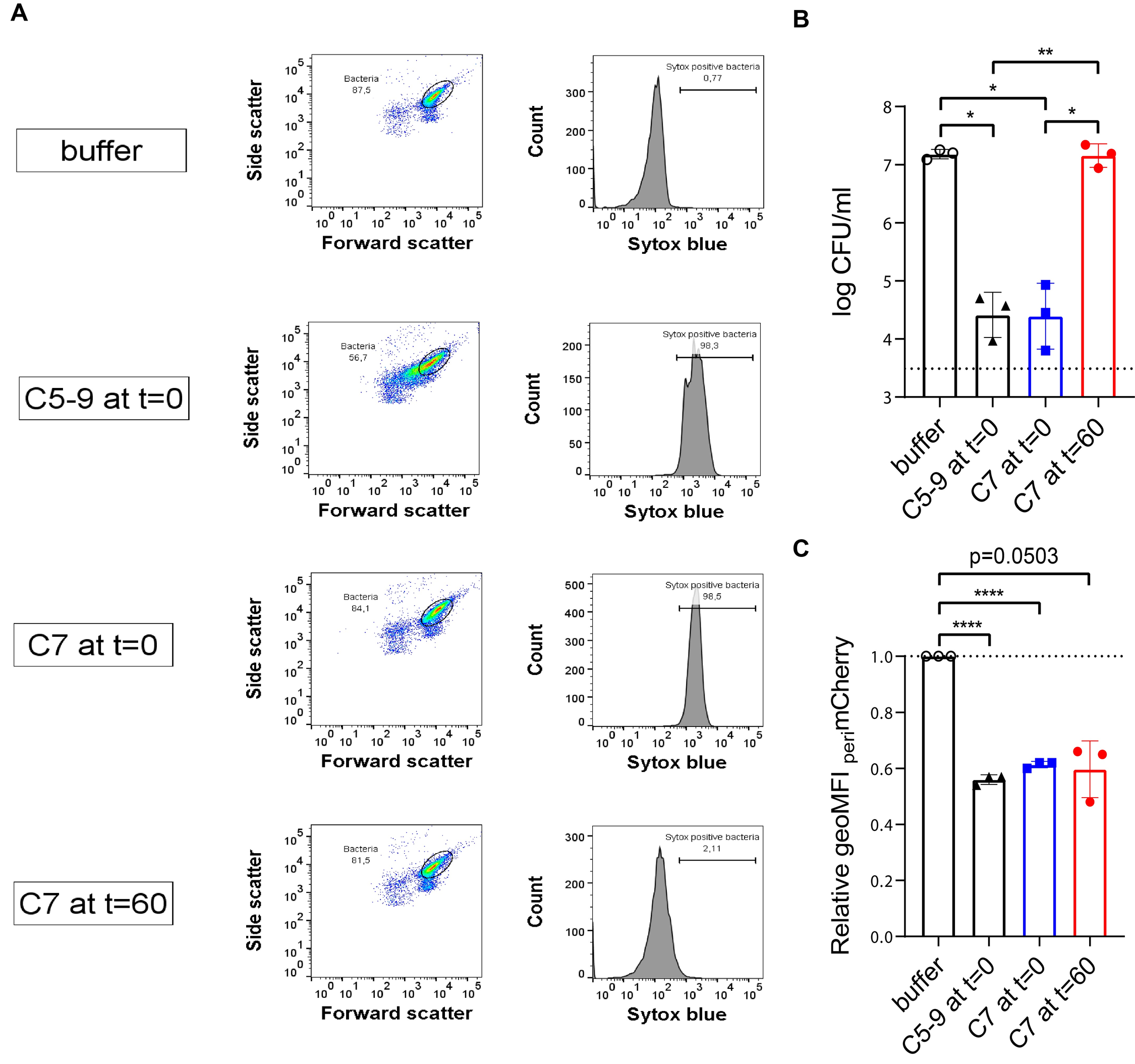

Supplement: S1 Fig — (A) Gating strategy of bacteria analysed by flow cytometry. Bacteria were gated based on forward scatter and side scatter. Sytox positive cells were gated such that the buffer control (top right) had <1% positive cells. Representative plots showing convertase-labelled MG1655 incubated with buffer only, C5-9 at t = 0, C7 at t = 0 and C7 at t = 60 samples described in Fig 2B. (B) Colony forming units (CFU) were also counted at the end of the same assay for MG1655 but with OmCI added at t = 15 for all samples (as described in Fig 2C). In short, 20 μl droplets of a 125x, 1250x, 12500x and 125000x dilution of the reaction volume were plated in duplo on LB agar plates that were incubated overnight at 37 ºC. (C) Bacterial outer membrane (OM) damage was also measured by flow cytometry for samples as described above for (B) In short, OM damage was measured by determining the relative geoMFI of mCherry compared to a buffer treated sample on MG1655 transformed with pFCcGi containing a constitutively expressed periplasmic mCherry (perimCherry) previously used in [16]. Data represent mean +- SD (B and C) of at least 3 independent experiments. Statistical analysis was done using a paired one-way ANOVA with Tukey’s multiple comparisons’ test. Significance was shown as * p ≤ 0.05, ** p ≤ 0.01 or **** p ≤ 0.0001. (TIF) [file ppat.1008606.s001.tif]

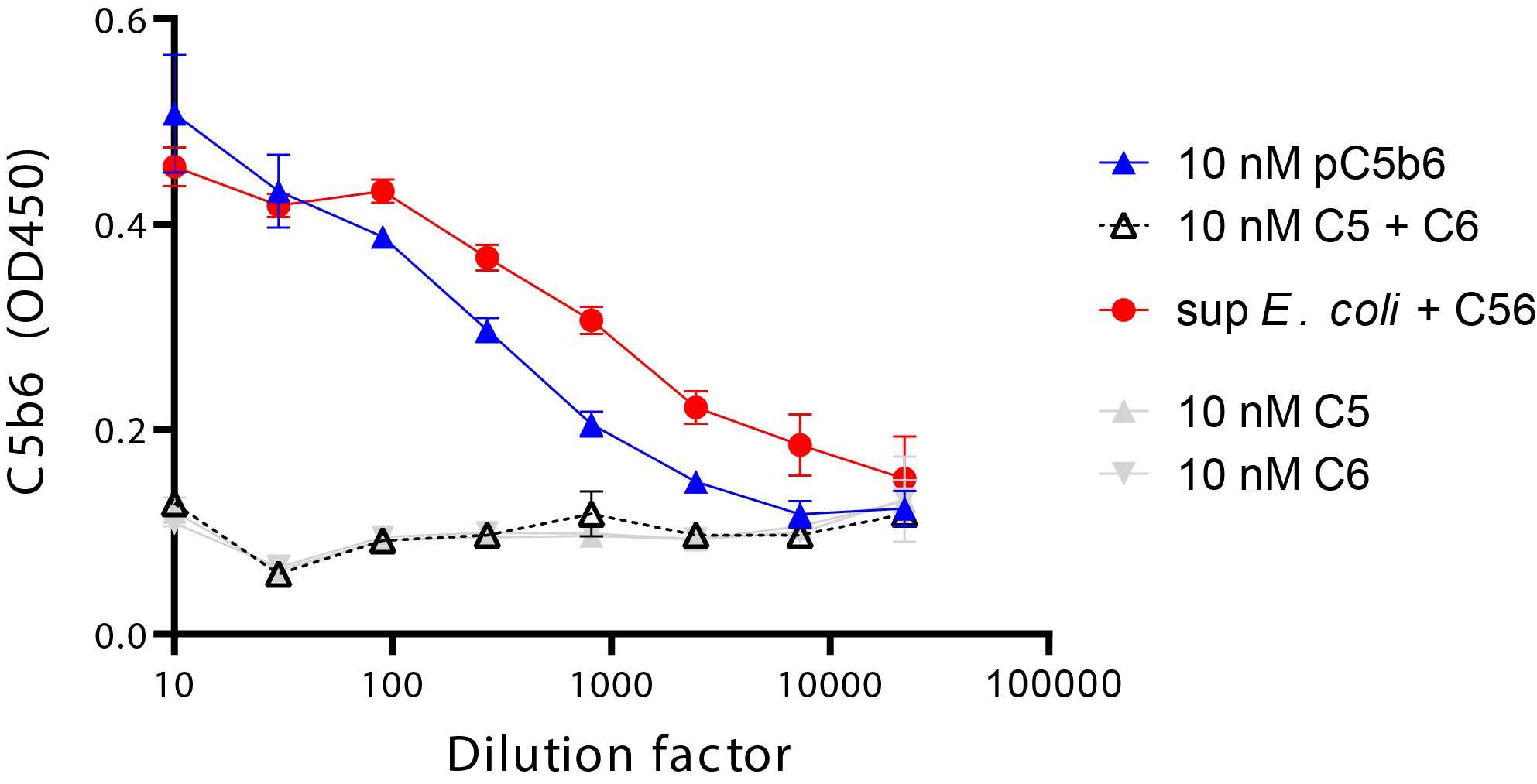

Supplement: S2 Fig — Specificity of the C5b6 ELISA is shown here. A titration of C5, C6, C5 + C6, purified C5b6 (pC5b6) or supernatant of convertase-labelled E. coli MG1655 incubated with C5 + C6 was added to ELISA plates coated with monoclonal anti-human C6 and next detected with polyclonal anti-C5. Data represent mean +- SD of at least 3 independent experiments. (TIF) [file ppat.1008606.s002.tif]

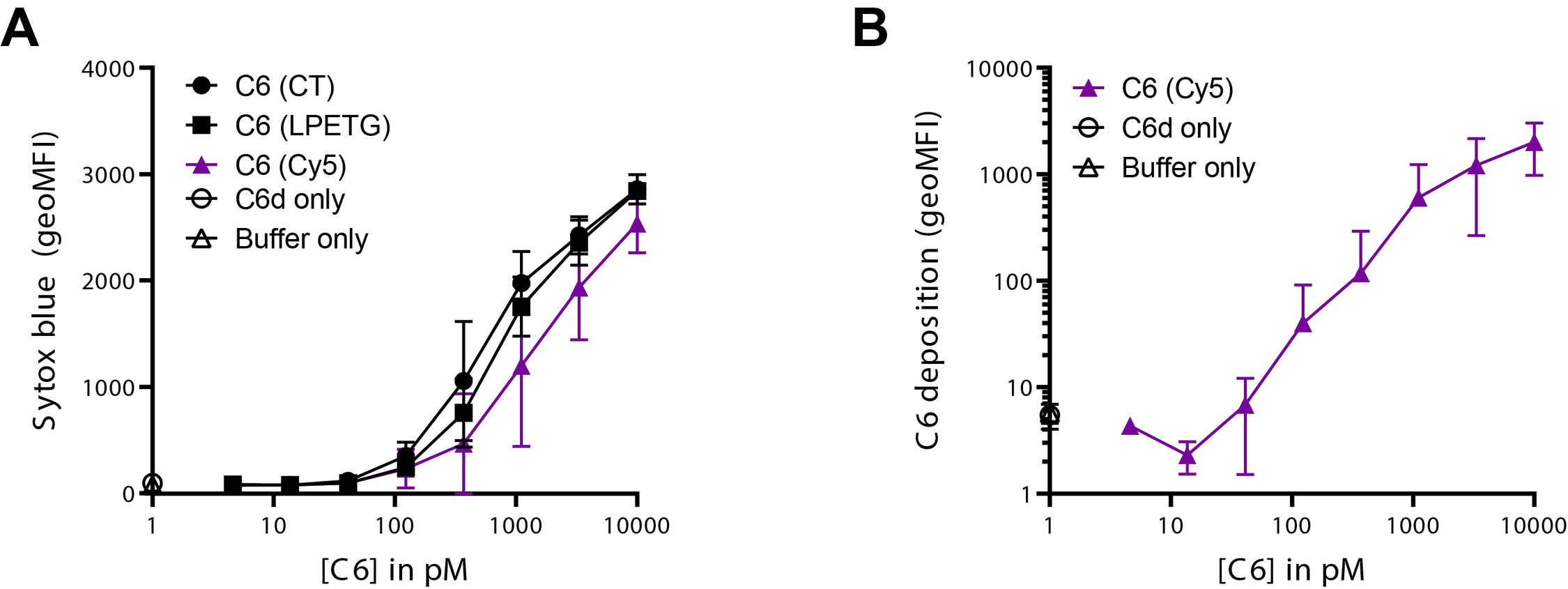

Supplement: S3 Fig — E. coli MG1655 bacteria were added to 1% C6-depleted serum supplemented with a titration of C6 isolated from plasma (CT = Complement Technology), recombinantly expressed C6-LPETG-His (LPETG) and sortagged C6-LPETGGGG-Cy5 (Cy5). (A) The percentage of bacteria with a damaged inner membrane as determined by Sytox staining. (B) Deposition of C6-Cy5 on bacteria was plotted as geoMFI of the bacterial population. Data represent mean +- SD of at least 3 independent experiments. (TIF) [file ppat.1008606.s003.tif]

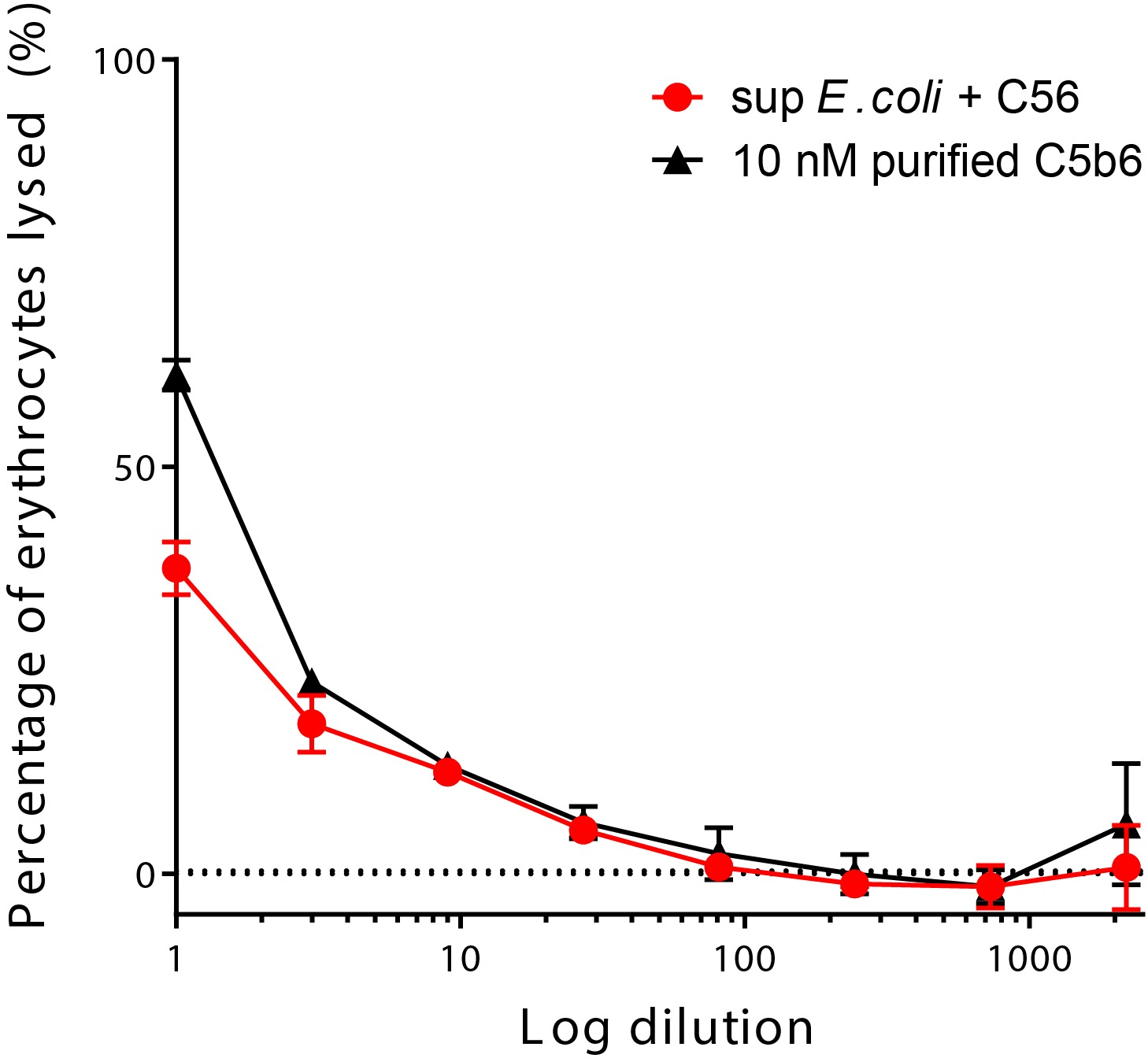

Supplement: S4 Fig — Rabbit erythrocytes were incubated with a titration of purified C5b6 (pC5b6) or supernatant of convertase-labelled E. coli MG1655 incubated with C5 + C6 in the presence of 10 nM C7, 10 nM C8 and 100 nM C9. The percentage of erythrocytes that were lysed was substracting background OD405 of erythrocytes in buffer (0% lysis) from each value and dividing this by the OD405 value of erythrocytes in MilliQ (100% lysis). Data represent mean +- SD of at least 3 independent experiments. (TIF) [file ppat.1008606.s004.tif]

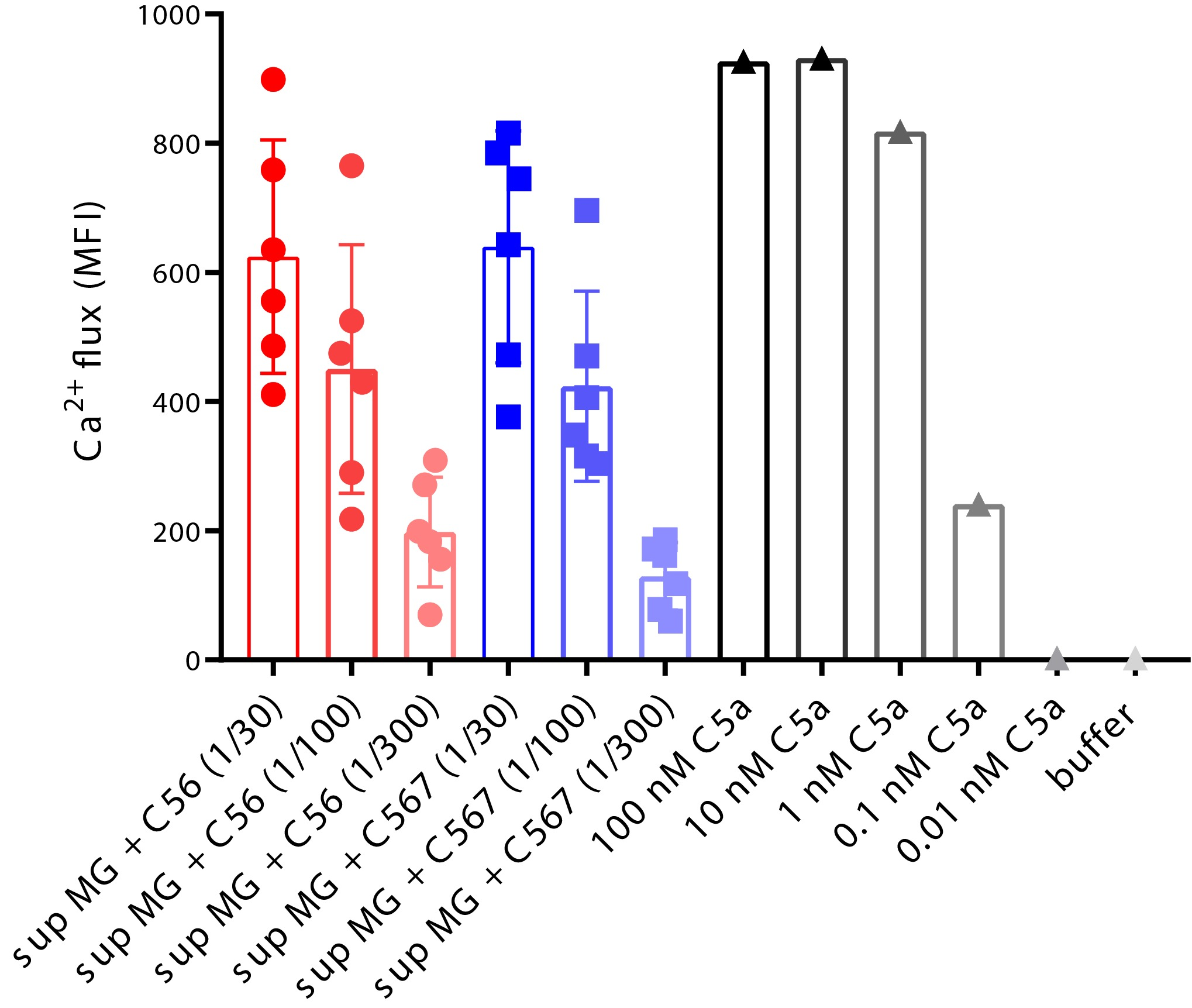

Supplement: S5 Fig — C5a was measured in the supernatant of convertase-labelled E. coli MG1655 incubated with 100 nM C5, 100 nM C6 in the absence (red circles) or presence (blue squares) of 100 nM C7 by a calcium flux-based reporter assay [55]. Supernatant was diluted 1/30, 1/100 and 1/300 times. A titration of purified C5a (filled triangles) was taken as standard. Data represent mean +- SD of at least 3 independent experiments. (TIF) [file ppat.1008606.s005.tif]

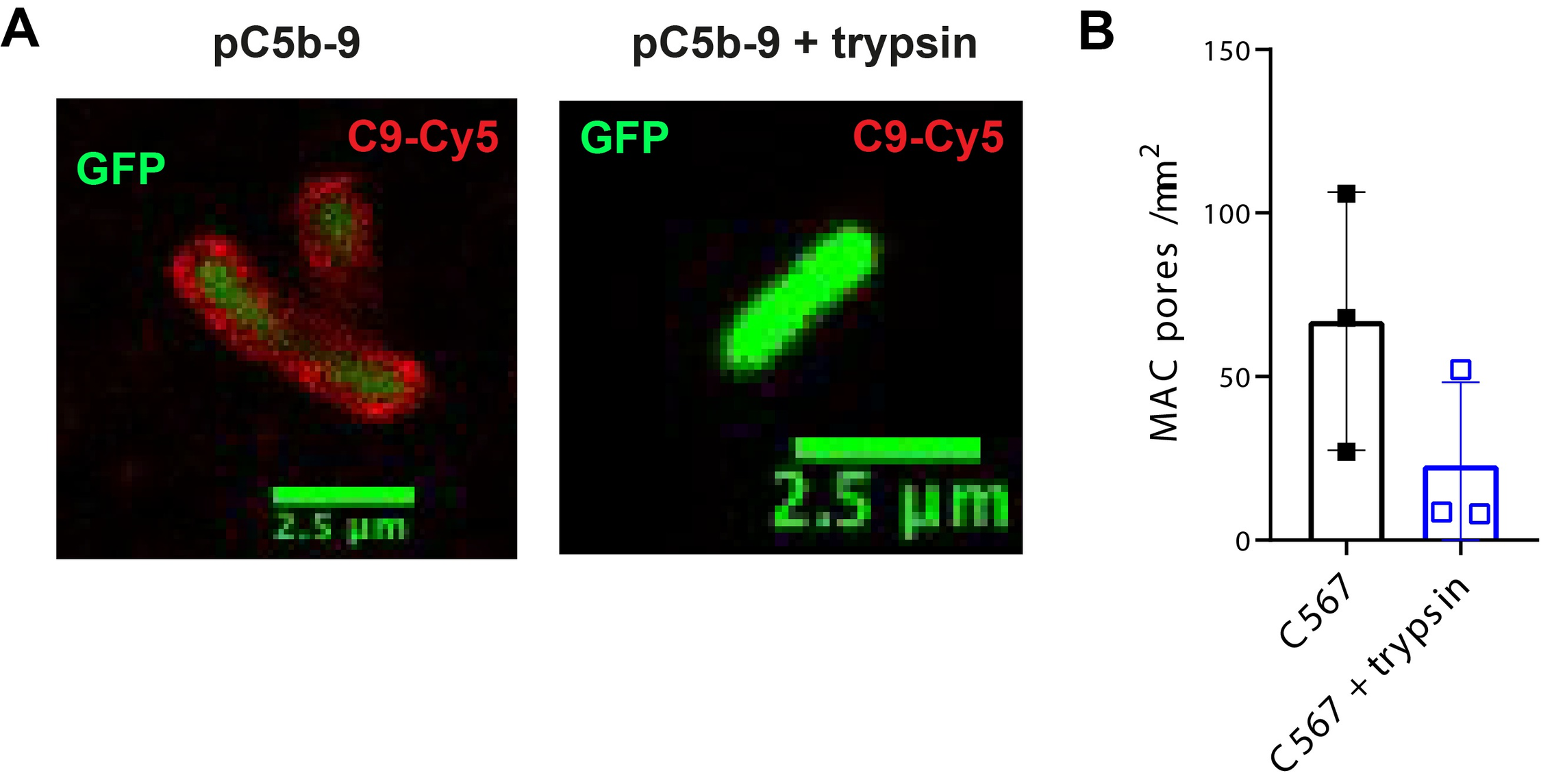

Supplement: S6 Fig — (A) GFP-induced E. coli MG1655 were immobilized on Cell tak (BD Diagnostics, USA) covered glass slides and next labelled with convertases with 10% C5-depleted serum. Next, bacteria were incubated with 100 nM pC5b6, 100 nM C7, 100 nM C8 and 1000 nM C9 and subsequently treated with buffer or 10 μg/ml trypsin. Samples were imaged using a Leica SP5 confocal microscope with a HCX PL APO CS 63x/1.40–0.60 OIL objective (Leica Microsystems, the Netherlands). (B) Quantification of MAC pores on atomic force microscopy images (phase images) of E. coli MG1655 immobilized on Vectabond covered glass slides and treated as in Fig 6D. The number of MACs per 500x500 nm2 scan was counted by hand and used to calculate the number of MACs per μm2 for each analyzed bacterium. Three bacteria were examined in each condition with at least four smaller scans per cell. (TIF) [file ppat.1008606.s006.tif]
