# Supplementary material for: Bacterial killing by complement requires direct anchoring of membrane attack complex precursor C5b-7
Source: PLoS Pathog. 2020 Jun 22;16(6):e1008606. doi: 10.1371/journal.ppat.1008606 (PMC7351214; doi:10.1371/journal.ppat.1008606)
Supplement: Attachment — Submitted filename: 102373_1_rebuttal_1695279_q6xl8z.pdf [file ppat.1008606.s007.pdf]

Authors' response to reviewers for manuscript "*Bacterial killing by complement requires direct anchoring of Membrane Attack Complex precursor C5b-7*" (reference #RC-2019-00125)

Our manuscript entitled "*Bacterial killing by complement requires direct anchoring of Membrane Attack Complex precursor C5b-7*" has been reviewed by Review Commons. We thank the referees for their interest in our study and are very pleased that the referees consider our findings novel, important and well-designed. Based on the comments given by the referees, we have revised our manuscript and have included two new experimental figures:

-Experimental data validating that our gating strategy with Sytox blue correlates well with bacterial killing on plate (**Fig S1-B**)

-Experimental data validating that non-bactericidal MAC complexes damage the bacterial OM (**Fig. S1-C**).

In the response letter below, we respond to the comments raised by the reviewers and explain how we have revised our paper accordingly. All changes into the revised manuscript are clearly highlighted in yellow.

POINT-TO-POINT REPLY

Reviewer #1

(Evidence, reproducibility and clarity (Required)):
The paper by Doorduijn et al. addresses a question rarely touched upon in modern studies of the complement system, namely the stability and time-resolved functions of complement component. It extends two earlier reports from the same laboratory, however, with a clear, novel point concerning especially the function of C7. The study embodies several techniques and modes of investigation. From these experiments, the paper contributes significantly to our understanding of the MAC complex is formed and why some bacteria escape this host defense mechanism. Over all the study is very well performed and written. I have only a few major comments.

Reviewer #1 raises 3 points:

POINT 1. *The AFM pictures shown in Fig. 6D are of outstanding quality. However, it is a disappointment that the outcome of complement incubation was shown only for a complement-resistant E. coli strain. Would it be possible to show the location on the bacterial surface of MAC complexes, or holes, on a complement-susceptible strains? Comparing the visual outcome for such bacteria with locally formed MAC versus C7 replenished would be quite interesting and perhaps important.*

ANSWER 1. Since **Fig. 6D** represents AFM images of MAC on complement-<u>susceptible</u> *E. coli* bacteria, we assume that the reviewer is asking why we did not perform AFM experiments on complement-resistant strains? To address this question, it is important to note that we have thus far not succeeded in robustly visualizing MAC complexes under conditions at which bacteria

were not killed by MAC complexes (Heesterbeek et al., EMBO J, 2019). While non-bactericidal MAC complexes are present on the bacterial surface as demonstrated with C9 deposition by flow cytometry, we hypothesize that they are not well inserted into the membrane (demonstrated by sensitivity to trypsin) and therefore difficult to resolve by AFM. This is consistent with previous AFM experiments on related pore-forming proteins (Leung et al, 2014, 2017), in which inserted pores were readily detected on supported lipid bilayers, but mobile, non-inserted pores were harder to resolve due to the invasiveness of the AFM measurement and/or insufficient temporal resolution. In the revised manuscript we now better clarify this in **line 298-301**.

POINT 2. *The flow cytometric analysis of bacterial killing is somewhat simplistic. Usually, staining of BOTH live and dead bacteria is performed. This permits better gating of the relevant populations. Specifically, the gating seems to fit the population in Fig. S1 only poorly, with the gate in some cases simply dividing what otherwise appears to uniform population ("C9 at t=0")*

ANSWER 2. In the revised manuscript, we have now included additional data demonstrating that our gating strategy with Sytox blue correlates well with bacterial killing on plate (new **Fig S1-B** referred to in **line 78-79**, **92-93**, **96-98** and **Supplementals text line 21-24** shows cfu data for Sytox data of **Fig 2D**). These data correspond with our earlier findings showing that cells gated to be positive for Sytox blue are indeed the relevant population of dead cells (Heesterbeek, EMBO J, 2019). We disagree with the reviewer that the use of a 'live' stain is of added value here. Because the outer membrane of Gram-negative bacteria is also a permeability barrier for 'live' stains like Syto9, MAC-dependent outer membrane perforation also results in increase in 'live' stain during the process of bacterial lysis (also described in Stiefel et al, BMC Microbiol, 2015 PMID: 25881030). We have therefore chosen to only use the Sytox stain in this study as this is a very reliable marker for killing.

POINT 3. *The cited literature is, in general, pertinent and comprehensive. I was surprised, however, that none of the many contributions to field of MAC formation by AF Esser was cited. For instance, the studies over C9 conformation (PMID: 2475785) seem not far away in topic from some of the points raised in the present paper.*

ANSWER 3. The reviewer is correct that the work of AF Esser has indeed focused on the contribution of C9 and C9 polymerization to the lytic activity of the MAC pore. In the revised manuscript, we have therefore now included some of the work done by AF Esser (**references 34, 36 and 37**) and have discussed this in our discussion (**line 305-309**). However, it is important to note that much of the work on the importance of C9 polymerization by AF Esser has been performed erythrocytes and single-membrane particles (also the suggested paper by the reviewer). Translation of these studies to the role of C9 conformation and polymerization on bacterial killing is therefore limited, although it does provide clues to what differences might cause the discrepancy observed between lysis of erythrocytes and bacterial killing by MAC pores.

Reviewer #1 *(Significance (Required)):*
Insight into the concept of locally formed MAC complexes is lacking and the paper clearly adds novel and quantitative data to this point. The paper probably mostly reaches out to an audience interested in the complement system and researchers interested in large protein complexes with conformational changes as part of their function. My own interest lies with complement-mediated protection against bacteria with a special focus on pattern recognition and protein-bacterial surface interactions.

<u>Reviewer #2</u>

(Evidence, reproducibility and clarity (Required)):
Doorduijn et al. present a study illustrating the importance of rapid C7 interaction with C5b6 for MAC-dependent killing of complement sensitive bacteria. The absence of direct C7 interaction results in a MAC which i) doesn't kill the bacteria, and ii) is sensitive to trypsin.

The authors have step by step investigated this issue by using common in vitro-methods with different strains of bacteria, serum, and/or purified complement proteins. Bacterial killing is evaluated by sytox blue influx in flow cytometry.

I like this work. The experimental strategy is sound, and the conclusions are convincing are based on the presented data. The data and the methods presented in such a way that they can be reproduced. I have no concerns regarding the design, execution or conclusions.

Reviewer #2 RAISES 3 POINTS

POINT 1. *My only criticism is on the number of replicates and following statistical analysis:*
• Overall, the experiments are conducted only three times. With the, in general, large differenced seen between the condition, this may still be acceptable. However, the statistic testing using only N=3 is of low value.

ANSWER 1. As the reviewer pointed out, with these *in vitro* studies where the experimental conditions are highly controlled it is common practice to perform three independent experiments when the differences are large.

POINT 2. *The authors have sometimes used paired testing, and sometimes unpaired. For example, Fig. 5A-B is based on paired testing, whereas data in C, which are based on A-B, is tested using unpaired testing. Why so is unclear to me.*

ANSWER 2. We thank the reviewer for this comment, as also for **Fig. 5C** we should have used a paired analysis, so we have done so accordingly in the revised manuscript (**line 725-726**).

POINT 3. *Further on in Fig. 5 A-B., ANOVA with Tukey multiple comparison tests is used, which implements testing between all conditions; still, only significance is reported for blue vs. red. If the intention was to only test red vs. blue, a t-test would be better.*

ANSWER 3. As with the previous comment, we have now performed a paired t-test since we only intended to compare C7 at t=0 vs C7 at t=60 in **Fig. 5A-B** (**line 725-726**). Moreover, for consistency in our statistical analyses we've also applied this to **Fig. 3C** (**line 706-707**).

Reviewer #2 *(Significance (Required)):*
As far as I understand, the presented data is of high significance for the conceptual understanding of the buildup of MAC for bacterial killing on Gram-negative bacteria.
I work partly with complement but is not an expert on the terminal pathway.

<u>Reviewer #3</u>

(Evidence, reproducibility and clarity (Required)):
The study is a follow-up on the paper the same group of scientists published in EMBO J last year. That paper showed that rapid interaction between C5b6 and C7 is necessary for effective

killing of Gram negative bacteria. The follow-up this paper makes is to make that case for a series of E. coli strains, showing as part of this that strains of clinical isolate E. coli resistant to complement attack prevent the rapid C5b6-C7 interaction. The story goes that C5 convertase engagement on the surface of targeted bacteria is the necessary context for effective C5>C5b conversion and thence interaction with C6 and C7. The rapid interaction with C7 is necessary because it prevents release/shedding of C5b6 from the bacterial cell surface. Overall, the conclusions seem justified - that C5b6 interaction with C7 stabilises its interaction with the surface and is needed to prevent C5b6 shedding. But this observation needs a mechanical or biophysical framework to be understood properly.

Reviewer #3 RAISES 5 POINTS

POINT 1. *The authors do not observe non-bactericidal MAC pores/non-lytic MAC by AFM and so I think in this study there is no evidence for their existence. Their depiction in Figure 8b is therefore misleading and I think should be deleted. Indeed, the authors do not know what the structure of the non-bactericidal MAC pores could be, so depicting them in this specific way isn't appropriate. They have no idea what they might be like, if they exist.*

ANSWER 1. We agree with the referee that we do not know the structure of a non-bactericidal MAC pore, and have therefore deleted the speculative structures in **Fig. 8B** (explained in **line 784-785**). Although we have no structural information, we do think that non-bactericidal MAC pores exist and our revised manuscript now includes new data to better explain this (**Fig S1-C**). While our initial manuscript showed that a delayed interaction between C5b6 and C7 results in MAC complexes that cannot perturb the bacterial inner membrane, we now show that these MAC complexes effectively damage the outer membrane (evidenced by leakage of mCherry from the periplasmic space (**Fig. S1-C,** explained in **line 97-98** and **Supplementals text line 24-31**). This leads us to conclude that there are pores formed in the outer membrane that are not capable of damaging the inner membrane. We think that within this context we can name these 'non-bactericidal MAC pores'.

POINT 2. *This brings me to another point: it is really unclear to me from this study how the authors envisage the inner bacterial membrane be damaged by MAC attack. Do MAC pores formed in the OM deliver MAC components to the IM? Or what happens - is the damage to the IM indirect? The reason why this is relevant to the possibility of non-bactericidal MAC pores is that it could be these are inserted just like bactericidal pores into the OM but the IM attack is deficient in some way.*

ANSWER 2. Although we agree with the reviewer that exact mechanism by which MAC pores perturb the inner membrane is unanswered, we think this is beyond the scope of this paper which mainly deals with the time-resolved functions of MAC assembly. However, to meet the referees' critique, we have now more clearly addressed this question in our discussion and speculate on several mechanisms by which the MAC pore could induce bacterial inner membrane damage (**line 277 - 288**). In short, we hypothesize that OM damage could indirectly trigger IM damage by affecting regulation of osmosis, overall cell envelope stability and/or envelope stress.

POINT 3. (Significance (Required)). *I am intrigued by the difference between MAC assembly on erythrocytes and bacteria. What do the authors believe to be the basis of this difference? It would help understanding of the significance of their work if they could make this clear. Without this kind of attempted explanation the results seem phenomenological - an observation has been made but why this observation occurs, what the important environmental difference is between erythrocyte membranes and the outer membranes of Gram negative bacteria is not*

addressed. I am looking for some kind of biophysical explanation - specific lipid properties, for example.

ANSWER 3. We agree that this is intriguing and in our revised manuscript we have included different hypotheses on why MAC assembly on erythrocytes and bacteria could be different. Although differences in composition between the erythrocyte membrane and outer membrane can definitely play a role, our data suggest that the difference is mainly a consequence of the fact that Gram-negative bacteria have two membranes (the outer and inner membrane). In the revised manuscript, the newly added figure (**Fig S1-C**) supports this, since this figure reveals that MAC pores generated from C5b6 that is generated in the absence of C7 can still damage the bacterial OM. However, despite observing OM damage by measuring leakage of a periplasmic protein, this does not lead to bacterial killing and IM damage. Since we here observe that rapid interaction between C5b6 and C7 is required for bacterial killing and IM damage, we think that efficient anchoring of C5b-7 is primarily relevant in damaging the bacterial IM and subsequently causing bacterial cell death. Finally, we have also mentioned this more specifically in the discussion of the revised manuscript (**line 277-288**).

POINT 4. *Related, at the end of the Results section the authors say "Altogether, these data indicate that complement-resistant E. coli can prevent complement-dependent killing by MAC pores by preventing efficient anchoring of C5b-234 7 and insertion of MAC pores into the bacterial cell envelope." My immediate response was: 'How? The Discussion needs to consider this.' But it doesn't.*

ANSWER 4. In the revised manuscript, we now explain this more extensively (**line 322 – 326**). In short, we hypothesize that the composition of the OM, mostly in terms of capsular polysaccharides and lipopolysaccharides, could affect this. We have added additional references supporting the role of these components in complement resistance in multiple Gram-negative species (**reference 45-48 and 50**).

POINT 5. *I was confused by the term "metastable lipophilic domain" at line 262 on page 10. Do the authors mean the MACPF domain?*

ANSWER 5. We have now more explicitly named this in our discussion as being the MACPF domain and have further elaborated what we meant by metastable (**line 263-267**).
