# Supplementary material for: Bacterial killing by complement requires direct anchoring of membrane attack complex precursor C5b-7
Source: PLoS Pathog. 2020 Jun 22;16(6):e1008606. doi: 10.1371/journal.ppat.1008606 (PMC7351214; doi:10.1371/journal.ppat.1008606)
Supplement: Peer review history

# Author comment: Author response to previous submission

**DOI:** 10.1371/journal.ppat.1008606.r001

2 Apr 2020

Attachment Submitted filename: 102373_1_rebuttal_1695279_q6xl8z.pdf Click here for additional data file.

# Editor report: Decision Letter 0

**DOI:** 10.1371/journal.ppat.1008606.r002

**Authors:** Guy Tran Van Nhieu, Dana J. Philpott

6 May 2020

Dear Dr. Rooijakkers,

We are pleased to inform you that your manuscript 'Bacterial killing by complement requires direct anchoring of Membrane Attack Complex precursor C5b-7' has been provisionally accepted for publication in PLOS Pathogens.

Before your manuscript can be formally accepted you will need to complete some formatting changes, which you will receive in a follow up email. A member of our team will be in touch with a set of requests.

Please note that your manuscript will not be scheduled for publication until you have made the required changes, so a swift response is appreciated.

IMPORTANT: The editorial review process is now complete. PLOS will only permit corrections to spelling, formatting or significant scientific errors from this point onwards. Requests for major changes, or any which affect the scientific understanding of your work, will cause delays to the publication date of your manuscript.

Should you, your institution's press office or the journal office choose to press release your paper, you will automatically be opted out of early publication. We ask that you notify us now if you or your institution is planning to press release the article. All press must be co-ordinated with PLOS.

Thank you again for supporting Open Access publishing; we are looking forward to publishing your work in PLOS Pathogens.

Best regards,

Dana J. Philpott

Associate Editor

PLOS Pathogens

Guy Tran Van Nhieu

Section Editor

PLOS Pathogens

Kasturi Haldar

Editor-in-Chief

PLOS Pathogens

​orcid.org/0000-0001-5065-158X

Michael Malim

Editor-in-Chief

PLOS Pathogens

orcid.org/0000-0002-7699-2064

***********************************************************

Thank you very much for submitting your work to PLOS Pathogens. I apologize for the delay getting the decision back to you. Overall the reviewers were very positive with the findings and I am satisfied with the way you addressed their (mostly minor) concerns. Congratulations on your nice work.

Reviewer Comments (if any, and for reference):

# Editor report: Acceptance letter

**DOI:** 10.1371/journal.ppat.1008606.r003

**Authors:** Guy Tran Van Nhieu, Dana J. Philpott

12 Jun 2020

Dear Dr. Rooijakkers,

We are delighted to inform you that your manuscript, "Bacterial killing by complement requires direct anchoring of Membrane Attack Complex precursor C5b-7," has been formally accepted for publication in PLOS Pathogens.

We have now passed your article onto the PLOS Production Department who will complete the rest of the pre-publication process. All authors will receive a confirmation email upon publication.

The corresponding author will soon be receiving a typeset proof for review, to ensure errors have not been introduced during production. Please review the PDF proof of your manuscript carefully, as this is the last chance to correct any scientific or type-setting errors. Please note that major changes, or those which affect the scientific understanding of the work, will likely cause delays to the publication date of your manuscript. Note: Proofs for Front Matter articles (Pearls, Reviews, Opinions, etc...) are generated on a different schedule and may not be made available as quickly.

Soon after your final files are uploaded, the early version of your manuscript, if you opted to have an early version of your article, will be published online. The date of the early version will be your article's publication date. The final article will be published to the same URL, and all versions of the paper will be accessible to readers.

Thank you again for supporting open-access publishing; we are looking forward to publishing your work in PLOS Pathogens. 

Best regards,

Kasturi Haldar

Editor-in-Chief

PLOS Pathogens

​orcid.org/0000-0001-5065-158X

Michael Malim

Editor-in-Chief

PLOS Pathogens

orcid.org/0000-0002-7699-2064
